# Impact assessment of the Centre for Research Excellence in Stroke Rehabilitation and Brain Recovery

**DOI:** 10.1186/s12961-023-00974-y

**Published:** 2023-05-01

**Authors:** Shanthi Ramanathan, Elizabeth Lynch, Julie Bernhardt, Michael Nilsson, Dominique A. Cadilhac, Leeanne Carey, Sandy Middleton, Jan Chamberlain, Frederick Rohan Walker, Penny Reeves, Andrew Searles

**Affiliations:** 1 Health Research Economics, Hunter Medical Research Institute, Locked Bag 100, New Lambton Heights, NSW 2305 Australia; 2 School of Medicine and Public Health, College of Health, Medicine and Wellbeing, University of Newcastle, Callaghan, NSW Australia; 3 The Florey Institute of Neuroscience and Mental Health, Melbourne University, Parkville, VIC Australia; 4 NHMRC Centre of Research Excellence in Stroke Rehabilitation and Brain Recovery, Heidelberg, VIC Australia; 5 Stroke and Ageing Research, School of Clinical Sciences at Monash Health, Monash University, Clayton, VIC Australia; 6 Occupational Therapy, School of Allied Health, Human Services and Sport, LaTrobe University, Bundoora, VIC Australia; 7 Nursing Research Institute, St Vincent’s Health Australia (Sydney) and Australian Catholic University, Darlinghurst, Sydney, Australia; 8 College of Nursing and Health Sciences, Flinders University, Bedford Park, SA Australia

**Keywords:** Impact assessment, Research translation, Stroke rehabilitation, Health economics

## Abstract

**Background:**

Research impact is an emerging measure of research achievement alongside traditional academic outputs such as publications. We present the results of applying the Framework to Assess the Impact from Translational health research (FAIT) to the Centre for Research Excellence (CRE) in Stroke Rehabilitation and Brain Recovery (CRE-Stroke, 2014–2019) and report on the feasibility and lessons from the application of FAIT to a CRE rather than a discrete research project.

**Methods:**

Data were gathered via online surveys, in-depth interviews, document analysis and review of relevant websites/databases to report on the three major FAIT methods: the modified Payback Framework, an assessment of costs against monetized consequences, and a narrative account of the impact generated from CRE-Stroke activities. FAIT was applied during the last 4 years of CRE-Stroke operation.

**Results:**

With an economic investment of AU$ 3.9 million over 5 years, CRE-Stroke delivered a return on investment that included AU$ 18.8 million in leveraged grants, fellowships and consultancies. Collectively, CRE-Stroke members produced 354 publications that were accessed 470,000 times and cited over 7220 times. CRE-Stroke supported 26 PhDs, 39 postdocs and seven novice clinician researchers. There were 59 capacity-building events benefiting 744 individuals including policy-makers and consumers. CRE-Stroke created research infrastructure (including a research register of stroke survivors and a brain biobank), and its global leadership produced international consensus recommendations to influence the stroke research landscape worldwide. Members contributed to the Australian Living Stroke Guidelines: four researchers’ outputs were directly referenced. Based only on the consequences that could be monetized, CRE-Stroke returned AU$ 4.82 for every dollar invested in the CRE.

**Conclusion:**

This case example in the developing field of impact assessment illustrates how researchers can use evidence to demonstrate and report the impact of and returns on research investment. The prospective application of FAIT by a dedicated research impact team demonstrated impact in broad categories of knowledge-gain, capacity-building, new infrastructure, input to policy and economic benefits. The methods can be used by other research teams to provide comprehensive evidence to governments and other research funders about what has been generated from their research investment but requires dedicated resources to complete.

**Supplementary Information:**

The online version contains supplementary material available at 10.1186/s12961-023-00974-y.

## Background

Stroke is a leading cause of death and disability worldwide [1] In Australia, a country of 25.7 million people, there were an estimated 445,000 people living with stroke in 2020 [2]. The total financial cost of stroke in Australia is estimated to be 6.2 billion Australian dollars (AU$) each year, with a further AU$ 26.0 billion associated with disability and premature death.

Recognizing a need to expand the evidence base for rehabilitation interventions, improve recovery and reduce the burden of disease for stroke, the National Health and Medical Research Council (NHMRC) of Australia funded the Centre for Research Excellence (CRE) in Stroke Rehabilitation and Brain Recovery (hereafter referred to as CRE-Stroke) from 2014 to 2019. The NHMRC CRE scheme provides support for teams of researchers to pursue collaborative research, with particular emphasis on building research capacity [3]. The vision for CRE-Stroke, an interdisciplinary and multicomponent programme for research and capacity-building, was to transform the stroke rehabilitation research and practice landscape in Australia and accelerate the development, translation and implementation of new interventions that are strongly supported by neuroscience [4].

There is a growing demand in Australia and globally for more accountability in public spending across all sectors, including health and medical research [5]. Despite this, impact assessment beyond academic outputs such as peer-reviewed publication citations is still not standard practice in many countries [6].

CRE-Stroke employed a team to apply the Framework to Assess the Impact from Translational health research (FAIT) [7] to (i) encourage research translation and optimize the impact of CRE-Stroke, (ii) assess its impact after the 5-year funding period, (iii) have greater transparency and accountability for the research investment made by the NHMRC and (iv) assess the feasibility and learnings from applying FAIT. FAIT is a hybrid of three proven methodologies for measuring research impact, namely, quantified metrics, economic analysis and narratives of the process by which research translates and generates impact. Details about the development of FAIT can be found in the seminal FAIT paper [7].

There is a plethora of impact assessment frameworks available including two recent systematic reviews of these frameworks, models and applications [8–10].

FAIT was selected as the preferred framework due to its multidimensional lens on impact, its ability to be prospectively applied, its flexibility and the opportunity to trial its feasibility for application to a research collaborative. Given there are no impact frameworks designed specifically for research collaboratives, we believed that FAIT was the best approach for the purpose of conducting a research impact assessment of a health-related research collaborative.

As described in the protocol paper [11], we initially planned to apply FAIT separately to the five nominated “streams of research” within CRE-Stroke (basic science, imaging, clinical trials, implementation science and data linkage). When applying the FAIT model to the streams of research, we recognized that there was considerable overlap in outputs, outcomes and impacts across the five streams. Moreover, we realized that a key measure of success of CREs is the ability to foster multidisciplinary collaborative research, and that many investments and capacity-strengthening activities funded by CRE-Stroke were directed at an “all-of-CRE” level. Consequently, we consulted with CRE-Stroke leaders and decided to undertake the impact assessment for the CRE as a whole.

We present the results from applying FAIT to CRE-Stroke and report on the feasibility and lessons from its application to a research collaborative/network rather than a specific project or programme.

The revised aim that specifically applies to our paper is to assess and report on the impact of CRE-Stroke as a research collaborative. In addition, we consider the feasibility of using FAIT’s package of validated impact assessment methodologies on an interdisciplinary research collaborative in stroke rehabilitation to inform future application.

## Methods

The setting was CRE-Stroke which brought together an interdisciplinary team of researchers primarily from two major stroke research centres in Australia: the Florey Institute of Neuroscience and Mental Health in Melbourne, Victoria, and the Hunter Medical Research Institute in Newcastle, New South Wales. Associate researchers and affiliates of CRE-Stroke are based at other sites across Australia. Funding for CRE-Stroke was AU$ 2.5 million over 5 years (2014–2019). The impact evaluation was coordinated by researchers (impact specialists and health economists) at Hunter Medical Research Institute who were separate from the stroke researchers that formed the leadership of CRE-Stroke. The impact evaluation received ethics approval from the University of Melbourne’s Human Research Ethics Committee (Ethics ID: 1647818.2).

### FAIT programme logic model

In year 2, FAIT programme logic models (PLMs) were developed for each of CRE-Stroke’s five research streams, which involved identifying needs, end users and impacts of each stream. Through a 6-monthly process of monitoring and feedback, teams from each stream had the opportunity to assess how they were tracking against their planned activities, outputs and intended impacts, to provide evidence of achievement of process, output and impact goals and to refine their research translation and engagement activities to maximize impact.

**Fig. 1:**
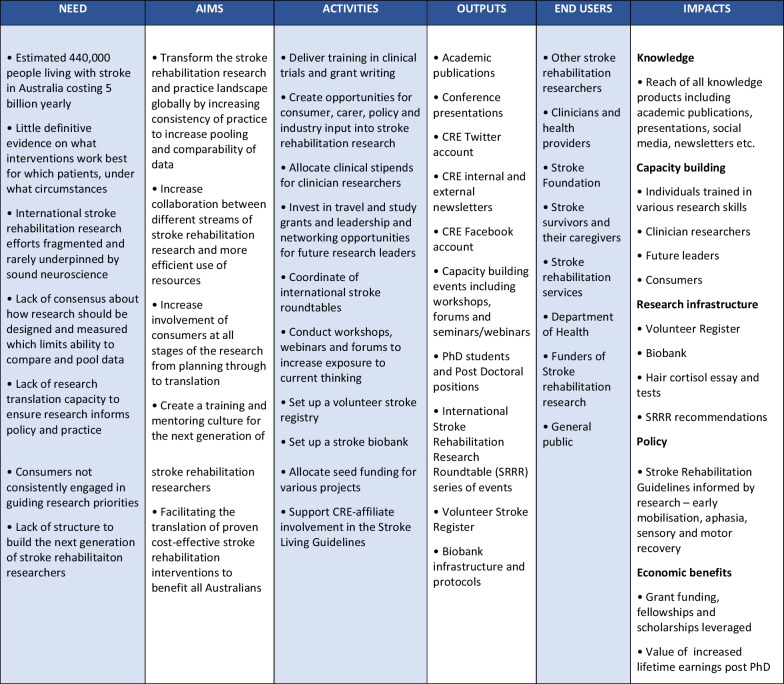
Modified programme logic model for CRE-stroke

The five streams’ PLMs were subsequently combined into an overarching CRE-Stroke PLM, collating work that was specifically funded, supported and/or enhanced by the existence of CRE-Stroke. This included an extensive range of capacity-building activities and investments. Figure 1 presents the combined CRE-Stroke PLM which was used to guide the impact assessment using FAIT.

We obtained data for the impact metrics via regular interviews and email communication with the management committee, stream leads, CRE-Stroke researchers and affiliates, CRE-Stroke administrative records including evaluations of CRE-Stroke events, an online survey of participants in CRE-Stroke activities, and phone interviews with consumers who were involved in CRE-Stroke activities. In addition, we searched relevant websites for citations and other usage statistics. A cutoff date of 10 November 2019 was used for all outputs and 1 August 2022 was used for publication statistics found on the Internet.

### Impact metrics: modified Payback

A modification of the Payback Framework [7, 12] was used to assess impact using quantitative metrics within the domains of knowledge advancement, clinical practice, policy and legislation, and economic impacts. Following discussions between the lead impact assessor (SR) and CRE-Stroke researchers, two additional domains of benefit “capacity-building” and “infrastructure” were included (with relevant metrics) to capture the infrastructure, education, training and professional development impacts of CRE-Stroke.

We reviewed the literature to identify existing impact indicators that could capture evidence of the anticipated benefits from the work of CRE-Stroke. The bulk of available indicators were designed for research programmes where the investment directly supported research activities. However, CRE-Stroke did not directly fund specific research projects apart from two discrete projects (one clinical trial and the development of a hair cortisol test). CREs are predominantly focused on capacity-building and translation of existing funded research projects. Hence, many indicators from the literature were not directly applicable to the purpose of the CRE research investment. This led to the CRE-Stroke stream leads identifying metrics that were relevant, measurable and timely for reporting the impacts of CRE-Stroke. Given the lack of indicators designed to measure investments in research infrastructure support such as supporting collaboration, capacity- and leadership-building and research translation, additional customized metrics were developed by the lead impact assessor in collaboration with the CRE-Stroke Management Committee to capture the anticipated impacts of the CRE-Stroke network and funded activities.

### Economic assessment

In the economic assessment, we compared the costs of delivering the CRE-Stroke programme of work to a calculated (monetized) value for the consequences, generated by that investment to provide an estimate of return on investment (ROI). Given that CRE-Stroke was focused on capacity-building and translation, and given the range and diversity of research outputs and key stakeholders for the CRE, the most appropriate and relevant method of economic analysis was a cost–consequence analysis (CCA). A CCA presents an array of consequences and costs in a disaggregated form [13]. The analysis aims to present a ledger of cost against a suite of attributable consequences, where some consequences are monetized and others are not. Our CCA only presents monetizable consequences, as the non-monetizable consequences are captured in the Payback metrics. We also present a conservative cost-to-consequence ratio—conservative because only a few of the consequences were meaningfully able to be monetized. Hence, the verdict of whether the investment in CRE-Stroke would be considered a good use of resources is left to the reader or decision-maker. A key question that informed attribution and what was included in the CCA was “Would this have happened without CRE-Stroke?” If the consequences of individual research and implementation projects, funded by other sources, would have occurred anyway without CRE-Stroke, then they were not claimed as a consequence of CRE-Stroke.

#### Direct and indirect CRE-Stroke costs

Both *direct* and *indirect* costs are based on opportunity cost. We captured direct CRE-Stroke costs from the CRE-Stroke research application and disaggregated them to provide greater transparency to the expenditure of funds on various activities.

Indirect CRE-Stroke costs were calculated via in-kind contributions from chief and associate investigators and were costed using a bottom-up micro-costing methodology [14]. The CRE-Stroke lead investigators (chief investigator A [CIA] and CIB) were assessed separately. We costed the salary for the CRE-Stroke CIA for 1 day a week (0.2 full-time equivalent [FTE]) and the CIB for a quarter of a day per week (0.05) to cover their respective contributions to the leadership and management of CRE-Stroke. The CIA and CIB wage was costed at a level E (professor) level. All other investigators were costed based on the time spent in CRE-Stroke Management Committee sessions and other mandatory CRE-Stroke activities. A schedule of meetings was cross-referenced with attendance data in meeting minutes to estimate the actual time each person was involved in CRE-Stroke teleconferences, workshops and events. Academic wages were costed using the Australian National University academic staff salary schedule [15] because there was no national noninstitutional academic staff salary schedule available in Australia. Investigator time was costed at a level D academic given that most investigators were employed as associate professors or professors. Wages for nonacademic investigators were costed using the average wage rate for Australia as a proxy [16]. An additional 21% was added to cover oncosts [15] and 27% to cover overheads. All costs were converted into 2021 values using standardized Reserve Bank of Australia inflation rates [17].

#### Implementation costs

We used similar bottom-up micro-costing methodology based on the opportunity cost for participants’ time and administrative records of the activities to estimate implementation costs for delivering the capacity-building activities offered by CRE-Stroke. The time allocation of each capacity-building activity was reported in hours and multiplied by the number of participants involved. The total number of hours was then divided into three groupings based on the work role of attendees (academics: 45%, clinicians: 35%, consumers and others: 20%). Academic time was costed at a level C senior lecturer, [18] and clinician time was costed at the average senior clinician wage for Australia [19]; the average wage rate for Australia was used for consumers and others [16]. CRE-Stroke initiatives also included research infrastructure (e.g. Stroke Research Register, Brain Biobank), coordination of international research collaborations (Stroke Recovery and Rehabilitation Roundtables [SRRR]), and contributions to policy (e.g. involvement in Living Stroke Guidelines). We used this same methodology to calculate the opportunity cost of attendance at the SRRRs. Given the difficulties with estimating potential travel and accommodation costs, we excluded these from the calculations. Calculation of the implementation costs for the Stroke Research Register and the Brain Biobank were limited to resources allocated by CRE-Stroke. No implementation costs were calculated for representation on the Living Stroke Guidelines project, which was coordinated by the Stroke Foundation, and would have happened without CRE-Stroke.

#### Consequences

The consequences of the investment in CRE-Stroke and its activities were already broadly captured using the Payback methodology. As part of the CCA, we monetized two key consequences of CRE-Stroke, the value of grants and fellowships awarded to CRE-Stroke affiliates, and savings from the Stroke Research Register. For the grants and other funds leveraged, individual recipients were asked to rate, on a scale of 0–100%, the contribution that their involvement in CRE-Stroke had on their ability to attract the funding in question. We used these estimates to value the research grants, consultancies, fellowships, scholarships and awards that could legitimately be attributed to CRE-Stroke. For the Stroke Research Register, we estimated the time taken to set up the register versus the time saved from subsequent recruitment for trials using records kept by researchers for timing of recruitment activities. These two examples are not direct outputs of research. However, given that the objective of the Australian government-funded CRE scheme is to “pursue collaborative research and build research capacity”, these examples are valid consequences for CRE-Stroke. The grants and fellowships in stroke rehabilitation that the CRE contributed to are a return on the research investment, ensuring a pipeline of strong, innovative, translational, consumer-informed, multidisciplinary, collaborative stroke rehabilitation research projects in the future. They also led to uplift and translation of research due to the increased capacity-building for recipients in implementation and impact. Similarly, the Stroke Research Register created a cost-effective alternative to direct recruitment for clinical trials and built research capacity in stroke rehabilitation research, another valid consequence of CRE-Stroke. The assumptions underpinning the analysis are listed in the results of the CCA.

### Narrative

To inform the narrative of the impact of CRE-Stroke, in May 2019 we emailed an invitation to all CRE-Stroke researchers and affiliates who had attended at least one event coordinated by CRE-Stroke, inviting them to participate in an online survey about research impact. The survey comprised a combination of closed- and open-ended questions and was administered by Qualtrics (for full survey please refer to Additional file 1: Research impact survey questionnaire). We asked participants about their experiences with CRE-Stroke, how research was translated and how research impact was generated both for their individual projects and for CRE-Stroke as a whole. We emailed a reminder invitation to all potential participants in July 2019. We also conducted individual semi-structured telephone interviews with stroke survivors to understand the impact of their involvement in CRE-Stroke. We collated all correspondence between affiliates and the CRE-Stroke coordination centre regarding research impact, along with evaluation forms from CRE-Stroke activities. We analysed quantitative data from the survey using descriptive statistics through Microsoft Excel. We thematically analysed the qualitative data from open-ended responses, interviews, evaluations and correspondence, and the results were used to inform impacts described in the narrative.

## Results

The impact results are presented against each of the three methods of impact assessment that make up FAIT. The impact survey was sent to 550 CRE-Stroke researchers and affiliates who had attended at least one event coordinated by CRE-Stroke. Of these, 110 responded, giving a response rate of 20%. Respondents reflected the makeup of the CRE: 70% were female, 70% were working in metropolitan regions, 78% were academics, clinician researchers or students, and 47% had been involved with the CRE for three or more years.

### Payback metrics

Table 1 presents the results from the application of the modified Payback method of assessment. The results are grouped under broad domains of benefit including knowledge advancement, capacity- and capability-building, infrastructure, policy and legislation, and economic benefit. Within the domain of *knowledge advancement*, CRE-Stroke associated peer-reviewed academic publications were cited over 7220 times. CRE-Stroke affiliates delivered over 620 oral and poster presentations at national and international conferences during the period 2014–2019. The SRRRs generated 10 unique publications, and by 18 May 2022 had been collectively downloaded over 129,000 times and received over 1350 citations. CRE-Stroke shared knowledge via internal and external newsletters with a mailing list that grew to almost 400 individuals representing academia, policy, industry and consumers and had an active presence on social media (2778 followers on Twitter, 328 on Facebook).

**Table 1:** Payback metrics scorecard

Domain	Subcategory	Impact metrics
Knowledge advancement	Peer-reviewed publications	354 publications through the end of 2019 (output metric)
		7224 citations, average Field-Weighted Citation Impact: 1.86
		Average 20.4 citations per paper
		470,243 times accessed including views and downloads
		70% open access
		4933 combined Altmetric score, average 16.4 per article
	Conferences	624 presentations overall
		503 oral presentations
		121 poster presentations
		40% with an international audience, 60% national/regional/local
		205 invited presentations
		116 unique presenters
	Stroke Recovery and Rehabilitation Roundtable	10 publications, *International Journal of Stroke* (5); *Neurorehabilitation and Neural Repair* (5) (output)
		1350 Crossref citations
		1289 Scopus citations
		129,118 downloads
	Engagement (internal & external)	393 newsletter subscribers
		31 CRE newsletters
		16 network newsletters (external)
		2778 Twitter followers
		3443 tweets
		32 unique Twitter curators
		328 Facebook followers and 304 likes
		59 examples of knowledge advancement in an impact survey of CRE affiliates
	Media interviews	2 Radio National interviews with Norman Swan (estimated listening audience 600,000)
Capacity- and capability-building	Supporting postgraduate, clinical and postdoctoral researchers	26 PhD students, 81% female
		6 PhD students funded directly
		39 postdocs, 67% female
		9 postdocs funded directly
		9 travel/study/future leader grants awarded
		26 researchers formally mentored by senior CRE affiliates
		7 clinicians awarded clinical stipends embedded in research teams
	MIDAS (modafinil drug trial)	CRE supported phase 2 of a trial that, if successful in phase 3, will help stroke survivors manage their fatigue
	Capability/capacity-building events	59 individual events
		32 seminars including CRE-affiliated research and international experts
		14 workshops on grant writing, clinical trials management, research translation and implementation, research impact and social media
		6 forums including consumer, industry and young stroke survivor forums
		4 early career researcher networking functions
		3 international Stroke Recovery and Rehabilitation Roundtables
	People whose capacity/capability was built	2126 individual occurrences of capacity/capability-building (47 events)
		2414 individual occurrences of capacity/capability-building (59 events, with 12 events based on projections of 24 attendees each—the mean number of participants at each event)
		2730 online occurrences of capacity/capability-building
		5166 total occurrences of capacity/capability-building
		9321 hours of capacity-building and training and education
		744 unique individuals who benefited
		68% female, 46% academics including students, 34% health services, 11% consumers, 9% industry/policy
		85 consumers involved in research prioritization, co-design and grant development
Infrastructure	Stroke volunteer register	650 active participants recruited in the Hunter Region of NSW
		13 studies have used the Register for recruitment
		8 studies completed, 3 ongoing
	Biobank	Physical facility set up and meets accreditation
		74 clots stored
		400 serum/plasma samples
		5 studies have samples stored but approval for cross-study usage still outstanding
	Stroke rehabilitation roundtables	Consensus-based recommendations for 5 areas of stroke rehabilitation research: (1) enhancing the alignment between preclinical and clinical research, (2) standardizing biomarkers of stroke recovery, (3) standardizing measurement of sensorimotor recovery in trials, (4) agreed definitions and shared vision for new standards in stroke recovery research, (5) improved development, monitoring and reporting of stroke rehab research
Policy and legislation	Living Stroke Guidelines	23 CRE affiliates on stoke rehabilitation working groups
		4 CRE affiliates on the implementation and evaluation working group
		4 CRE affiliates referenced in the guidelines
		1 CRE leader chairing the Stroke Rehabilitation Guidelines Working Group
	Engagement with policy-makers	2 Stroke Foundation representatives on CRE Management Committee
Economic	Grants and consultancies leveraged	148 grants and consultancies leveraged
	Fellowship leveraged	8 fellowships leveraged
	Scholarships and travel grants leveraged	11 scholarships or travel grants leveraged
	Awards leveraged	17 awards leveraged
	No. of PhD completions resulting in increased lifetime earnings	14 PhD completions

Within *capacity-building for research*, CRE-Stroke partially funded six PhD students, nine postdoctoral researchers and seven clinician researchers (the latter via clinical stipends to release them from clinical duties to build research skills). In addition, CRE-Stroke funded 59 capacity-building events such as workshops and consumer forums (see Appendix for full list of events), which were attended by over 744 unique individuals including 85 consumers and carers. Over the 5 years, CRE-Stroke was responsible for a total of 5144 individual capacity-building occasions including 2126 occasions based on actual attendance records, 288 based on a conservative estimate of 24 participants per session (mean attendance) for the 12 sessions for which attendance data were not available, and 2730 online attendances including downloading of recordings. This translated to approximately 9321 hours of building the research and translation knowledge, capacity and capability of future stroke research leaders, clinicians, and industry, policy and consumer representatives. Within the *infrastructure* domain, CRE-Stroke provided financial support to establish the Stroke Research Register and Brain Biobank to improve efficiencies in stroke rehabilitation research by minimizing the cost and time taken to recruit participants and obtain tissue samples.

Within *policy and legislation*, the most profound impact was the contribution to the Living Stroke Guidelines, managed by the Stroke Foundation of Australia. Twenty-three CRE-Stroke researchers were involved in various guideline working groups. The Rehabilitation Guidelines Working Group is chaired by a CRE-Stroke researcher, and four CRE-Stroke researchers are referenced directly in the guidelines. Within the *economic* domain of benefit, CRE-Stroke researchers secured over AU$ 18.8 million in research, fellowship and scholarship funding that was attributed by individual researchers as being a direct consequence of their involvement in CRE-Stroke. The total value was AU$ 68.3 million. Collectively, the impact of the CRE-Stroke using the Payback method is substantial.

### Economic analysis

Table 2 presents the cost–consequence tabulation of the economic benefits generated by CRE-Stroke. All consequences of CRE-Stroke are listed under the Payback results (Table 1). To minimize duplication, we have included only the consequences that can be monetized in the cost–consequence results. The cost of delivering the various research activities, research leadership, research support and capacity-building under the CRE-Stroke umbrella (including the AU$ 2.5 million direct investment by the NHMRC and an additional AU$ 409,094 worth of in-kind contribution from Australia’s leading stroke rehabilitation researchers) totalled AU$ 2.9 million over the 5 years. The implementation cost (opportunity cost) for attendance at CRE-Stroke capacity- and leadership-building events totalled AU$ 992,646, bringing the total investment to just over AU$ 3.9 million over the 5 years. Consequences from this investment that could be monetized and were deemed attributable to individuals’ involvement in CRE-Stroke totalled AU$ 18.8 million (sensitivity analysis (*S.A.*) *$13.7 million–$34.3 million*) indicating a return of $4.82 (*S.A. $3.50–$8.79*) for every dollar.

**Table 2:** Cost–consequence results

Costs	Description	AU$ 2020 value	
Direct CRE costs	Postdocs and future leaders	360,116	
	PhD scholarship contributions	129,924	
	Clinical stipends	120,000	
	Travel/study/future leader grants	108,000	
	Admin (salaries for CRE manager and admin support)	717,703	
	Impact assessment	139,341	
	Capacity-building events	125,614	
	Stroke Biobank	230,828	
	Modafinil in Debilitating Fatigue After Stroke (MiDAS)	195,216	
	Development of hair cortisol test	150,290	
	Hunter Volunteer Stroke Register	77,210	
	Service change and Supporting Lifestyle and Activity Modification after Transient Ischaemic Attack (SLAM-TIA)	72,472	
	Stroke Recovery and Rehabilitation Roundtables (SRRR)	62,116	
	Consultants	11,170	
	Total direct research cost	2,500,000	
Indirect CRE costs	In-kind labour CIA & CIB	366,541	
	In-kind labour other CI/associate investigators & non-funded associates	42,553	
	Total indirect research contribution	409,094	
Implementation costs	Attendance at capacity-building and other CRE events	992,646	
**Total CRE research and implementation costs**		**3,901,740**	

Consequences	AU$ 2020 value (attributed)	Lower (20%)	Upper (50%)
Grants leveraged—All grants and consultancies	17,411,114	12,995,130	32,487,825
Fellowships leveraged—All NHMRC and other fellowships	771,986	539,999	1,349,997
Scholarships leveraged—PhD scholarships and travel grants	162,302	38,360	189,552
Awards leveraged—Value of award monies	25,476	10,190	50,952
Increase in lifetime earnings—Completed PhDs (14) @ $30,000	420,000	84,000	210,000
Savings from usage of Hunter Stroke Register	Not monetized		
Savings from usage of Stroke Biobank samples	Not monetized		
Adoption of modafinil for managing post-stroke fatigue	Not monetized		
Commercialization potential of hair cortisol testing	Not monetized		
**Total return on investment**	**18,790,879**	**13,667,679**	**34,288,325**
**Return on investment (per dollar spent)**	**4.82**	**3.50**	**8.79**

This is a conservative estimate of ROI in CRE-Stroke given other potential, but as yet unvalued, consequences of CRE-Stroke investment not included in the analysis. These included potential savings because of investment in research infrastructure: (1) more streamlined recruitment to 13 projects using the Stroke Volunteer Register and (2) potential savings generated by the use of clot and serum/plasma samples from the Brain Biobank. Although CRE-Stroke did not fund research, it provided seed funding to support selected research that was deemed to be of significance to all stroke rehabilitation interventions including the management of stress and fatigue. Potential monetizable benefits from these investments not included in the CCA were (1) the potential commercialization consequences of the hair cortisol test and (2) potential monetary consequence of the approval of modafinil to treat post-stroke fatigue if the phase 3 trial (MiDAS2) is positive.

### Narrative

Table 3 presents the narrative of CRE-Stroke which summarizes the pathway to impact from need for a CRE in stroke rehabilitation through to its impacts, as depicted in the programme logic model (Fig. 1), and provides the context against which the results from the Payback and CCA can be interpreted.

**Table 3:** Narrative of CRE-Stroke

**Unmet need (as of 2014 when CRE-Stroke commenced)** There were an estimated 440,000 people living with stroke in Australia, costing almost AU$ 5 billion yearly to treat. There was little definitive evidence on what interventions work best for which patients, under what circumstances. International stroke rehabilitation research efforts were fragmented and rarely underpinned by sound neuroscience. Building evidence-based treatments for rehabilitation was a major global priority, requiring a coordinated effort at national and international levels. However, there was a lack of consensus about how research should be designed and measurement parameters within research studies (timing, use of consistent rehabilitation outcomes), limiting the ability to compare and pool data. There was also a lack of research translation capacity to extend stroke rehabilitation research into the future. Consumers were not consistently engaged in guiding research priorities, and there was a lack of structure to build the next generation of stroke rehabilitation researchers.
**Response** Initiatives were developed to address identified needs by transforming stroke rehabilitation research and practice, creating a training and mentoring culture for the next generation of stroke rehabilitation researchers, and facilitating the translation of proven cost-effective stroke rehabilitation interventions to benefit all Australians. CRE resources were spent on the following key activities: • training in clinical trials and grant writing • opportunities for consumer, carer, policy and industry input into stroke rehabilitation research • clinical stipends to build capacity amongst clinician researchers • professional development of the next generation of stroke rehabilitation researchers including travel and study grants and leadership and networking opportunities • coordination of international stroke roundtables to achieve consensus, collaboration and comparability across many aspects of stroke rehabilitation research • exposure to current thinking around research translation, implementation and impact through workshops, webinars and forums • setting up a volunteer stroke registry • setting up a stroke biobank • seed funding for various stroke rehabilitation research projects • supporting CRE-affiliate involvement in the Living Stroke Guidelines
**Outputs** • 354 stroke rehabilitation academic publications • 625 conference presentations (250 at international events) • 2778 followers of CRE Twitter account (32 unique curators, disseminated 3443 tweets) • 393 subscribers to CRE newsletters (31 external and 20 internal editions) • Coordinated 59 capacity-building events, attended by 788 affiliates including researchers, policy-makers and consumers, with 5144 individual occurrences and 9321 hours of capacity-building • Supported financially or in-kind 26 PhD students (81% female) and 39 postdoctoral fellows (69% female) and administered 7 clinical stipends • The Stroke Recovery and Rehabilitation Roundtable series of events produced 10 publications • The Stroke Research Register has over 600 registered stroke survivors available for clinical trials
**Impacts** Given the extensive evidence of impact obtained from the various data sources, the impact is reported in the main body of the paper both in the Payback metrics (Table1) and below

To fully capture the benefit of CRE-Stroke and include the views of its affiliates including stroke rehabilitation researchers, clinicians and consumers, the impacts are described in more detail here, by the domains of impact used in Table 1.

#### Knowledge advancement, capacity- and capability-building

Regular CRE-Stroke presentations and capacity-building activities have advanced knowledge about research practice amongst CRE-Stroke affiliates.

*I changed from using SF36 [36-Item Short Form Survey] assessment with patients to using the Fatigue Assessment Scale (FAS)—after hearing X’s presentation.*

*Learnt the importance of involving consumers (stroke survivors) more and with greater effect in my research at the design stage.*

*I have used this knowledge to write a number of grants including an NHMRC investigator grant application.*

*Used the knowledge to co-design a mobile application…….to connect with patients and family.*

And from the perspective of consumers:

*…I’ve really learnt the lesson that when I am presenting, I am not always right… Everyone’s got the right to have their own opinion, that’s what makes the world go around and that when I do present now, it’s not always about me. I remember that I’m the face of a community, I’m often the voice of a group [of stroke survivors] that can’t express themselves.*

The SRRRs produced international recommendations which provided global leadership and influence on the stroke research landscape that led to the standardization of stroke rehabilitation research. Some examples of local and international use of the Roundtable publications include the following:

*I’m writing about your thoughtful 2017 consensus article about standardizing measurements of sensorimotor recovery. I’ve used it to align my outcome measures for a forthcoming recovery study, and [a colleague] is also using it for a grant application.* [researcher, USA]

*We’re in process of evaluating the outcome measures we are currently using in the management of stroke patients, and we’re keen to align our outcome measures closely with the recommendations provided in the article written by Kwakkel et al. 2017…as we are looking to include some clinical research in the future.* [hospital-based physiotherapist, Australia]

*SRRR helped with determining guidelines for pre-clinical stroke research which is fundamental when designing a study.* [CRE-Stroke affiliate]

Capability-building in communications for CRE-Stroke researchers and affiliates resulted in greater visibility of stroke rehabilitation in both the media and social media.

*It really assisted me with setting up my Twitter profile and manuscript development (especially key word choice) and using Twitter to share new research and/ or research ideas. I have been able to recruit to my main PhD study as well through social media.*

It also led to stroke rehabilitation researchers building new partnerships and collaborations with research, clinical and consumer groups and reviewing the way they communicated and disseminated information from their research:

*I developed a small research working group with the consumer as the driver of my research proposal.*

*I have become part of a collaborative of early career researchers interested in the topic area of mild/minor stroke, and together we are planning a scoping review.*

Clinical stipends provided seven stroke rehabilitation clinicians with the opportunity to be embedded in research and transfer those skills back to their workplaces.

*The clinical stipend allowed me to spend time away from my clinical duties as a speech pathologist and immerse myself in research…I learnt research skills and knowledge about research methods I can take back to my workplace and hopefully be involved in more research in the future.*

Two grant writing workshops had positive impacts on writing grant applications, leading to several large grants that were attributed to the transfer of knowledge and skills.

*I refer to the suggestions of the expert presenters often when preparing grant applications now, including how to structure grant application responses and how to frame application content in the most appropriate way.*

*The grant writing workshop … was instrumental in changing the way I approach grant writing. A very valuable experience, with learnings that I continue to apply to my work. I was so pleased to get my first big grant as a CIA. Thank you CRE.*

Many CRE-Stroke future leaders have secured employment in academic institutions across Australia, Canada and Malaysia, further extending stroke rehabilitation research and implementation expertise.

### Policy change

In addition to CRE-Stroke substantial representation on the Stroke Foundation’s Living Stroke Guidelines and contribution to policy in areas such as sensory and upper limb rehabilitation, the phase 3 AVERT [A Very Early Rehabilitation Trial after stroke] trial has informed the potential dangers of early mobilization globally including removal of this practice from guidelines [20]. Consumers involved with CRE-Stroke also spoke about the opportunity to be involved in policy:

*Last year I was involved in an international, BMJ Rapid Recommendation and …I sat there and thought “Oh my goodness, I’ve just been involved in making recommendations that will affect policy change internationally.”*

### Research practice and infrastructure

Three CRE-Stroke-run clinical trials workshops transferred knowledge and experience from successful clinical trialists to assist junior trialists in planning and running effective and cost-efficient trials.

*Attending the Clinical Trials Workshop made a big difference to how I attempt to plan and design investigator-initiated studies. I consult my notes often and use checklists I developed when attempting to devise study protocols for new research projects. I also use these teachings to guide clinicians who are interested in starting a formal research project.*

*Much of the insights gained from the workshop have been translated into the clinical trial I am currently project managing. I can see improvements in my project management/coordination.*

The Stroke Research Register has 650 registrations and helped provide a good cross section of stroke survivors, saving researcher time and recruitment expenses on many clinical trials and studies.

*I would not have been able to complete my study without the help of the Stroke Register. We needed to get a good cross-section of stroke survivors and the Register was able to provide us with that diversity of patients. Of course, we could have tried to recruit ourselves, but it would have been an ineffective and inefficient use of researcher time. The Register staff also gave us outstanding support. Unlike other Registers, they didn’t just upload our study information, they worked with us to ensure the invitation was interpretable by patients on the Register.*

Consumers also benefited and saw the Register as a way of finding their community of others who had lived through stroke.

*There is nowhere really to go (after a stroke) except to the GP after stroke and it feels reassuring to be part of a professional organization.*

Cross-study usage of the Brain Biobank is being finalized and will further reduce the cost of stroke research projects. Hair cortisol testing, developed to look at the effects of stress on stroke recovery, has been translated to other sectors including the Australian Defence Force.

### Potential future clinical practice change

CRE-Stroke supported the phase 2 trial of modafinil (an insomnia drug showing a positive effect on post-stroke fatigue) that showed the potential to greatly improve the well-being of stroke survivors. Stroke survivors with non-resolving fatigue reported reduced fatigue and improved quality of life after taking 200 mg daily treatment with modafinil.

*In the results of the Phase II trial, supported by CRE-Stroke, we found that Modafinil did effectively alleviate fatigue and improve quality of life. Even more gratifying than the statistical results (which allowed us to obtain funding for a larger phase III clinical trial) were the individual stories from patients…. Three patients managed to return to work which had been impossible due their level of fatigue.*

The clinical trials stream, incorporating many existing trials, also continued to inform stroke rehabilitation practice. The phase 3 AVERT trial has informed the potential dangers of early mobilization globally [20] and was named by the Physiotherapy Evidence Database as one of the five most important trials published in the years 2014–2019, providing a large international stroke rehabilitation database for ongoing research and guiding practice internationally. The SENSe [Study of the Effectiveness of Neurorehabilitation on Sensation] trial has contributed knowledge on improving somatosensory recovery and arm use [21], the VERSE [Very Early Rehabilitation in Speech] trial is informing therapy selection for aphasia [22] and the BUST and other trials have contributed to informing therapy for motor recovery post-stroke [23]. Together, these trials have created evidence to guide stroke rehabilitation practice from early mobilization to aphasia, including evidence of what not to do.

### Community benefit

The bringing together of researchers, stroke survivors and their families has increased overall collaborations with consumers and improved stroke survivor engagement, empowerment and well-being.

*It has contributed to things like cohesiveness of families, like education, like there’s so much knowledge now about impact, about how that is delivered to families who are affected by a stroke. That’s what I am passionate about.*

*I felt very much like I had the opportunity to be involved, to participate and have a voice.*

*I felt very included in the bigger picture, whereas when everything [the stroke] happened, I just felt like I was a piece of meat….so that’s had enormous benefit, feeling included in the bigger picture.*

## Discussion

With an investment of AU$ 3.9 million over 5 years, CRE-Stroke delivered a conservative ROI estimate of AU$ 4.82 for every dollar invested. This is a substantial ROI from a funded NHMRC CRE. In addition, many CRE impacts such as capacity-building in key areas of research are listed as consequences of CRE-Stroke but not monetized. Also missing are the many potential downstream, and yet unvalued, impacts of investment in research infrastructure and seed funding of discrete groundbreaking research.

Collectively, CRE-Stroke members produced over 360 publications and delivered 624 presentations; CRE-Stroke supported 26 PhDs, 39 postdocs and seven novice clinician researchers, all potential future stroke research leaders. There were 59 capacity-building events benefiting 744 individuals including policy-makers and consumers. CRE-Stroke created research infrastructure that has far-reaching impacts for the conduct of future stroke rehabilitation (and other) research, including the research register of stroke survivors and a brain biobank. Its global leadership produced international consensus recommendations including a shared vision for new standards in stroke recovery research to influence the stroke research landscape. Members contributed to the Australian Living Stroke Guidelines, and outputs from four CRE-Stroke researchers were directly referenced.

CREs are a unique form of research investment that are designed to encourage research excellence through capacity-building, infrastructure and research translation. These collaboratives have not typically been evaluated based on their impact. Using CRE-Stroke as a case example, our study demonstrates that these capacity-building platforms do create substantial impact, and even when conservatively measured, CRE-Stroke’s benefits greatly outweighed its costs.

Rising public debt is increasing the need for accountability and transparency in how public money, including funded health and medical research, is returning benefit to the community. While our study focused on a publicly funded CRE, private philanthropies are also driven to understand how their research investments contribute to community health and well-being.

### Application of FAIT

Application of the mixed-methods framework to assess and support the impact of a research centre is novel, because while most existing impact assessment frameworks are retrospectively applied, FAIT was prospectively applied, during the life of CRE-Stroke. Applying FAIT to a CRE rather than a research project (as per its original intended use) was feasible. As per best practice for impact assessments, we customized the metrics to the planned activities, outputs and aspirational impacts of CRE-Stroke as reported in Fig. 1. Given that CREs must have a strong focus on capacity-building, research translation and infrastructure, and together with CRE-Stroke stream leads and key contributors, we developed indicators to capture these impacts for CRE-Stroke. Indicators of impact from these concepts are not widely used nor are they widely reported in the literature, so preliminary work with CRE-Stroke leaders was required to develop customized indicators that would collect evidence of the expected impacts. This practice is consistent with intended customization and previous applications of FAIT [24–26]. Apart from measuring whether an impact occurred, we also needed to determine whether the impact was fully or partially attributable to CRE-Stroke, which was not always clear (for instance when CRE-Stroke affiliates were awarded grants and reported CRE capacity-building activities had assisted them in writing their application). Most the research projects affiliated with CRE-Stroke were externally funded. We did not include the impacts of these research projects unless CRE-Stroke contributed to the translation of the research outcomes through to policy and practice. There were examples where this occurred—through the translation of findings from the AVERT trial. Through application of FAIT to a “research-enabling” funding vehicle, such as a CRE, we have identified the important methodological step of defining what is and is not attributable to a research collaboration.

We were able to collect relevant data to measure the impact of CRE-Stroke, but in addition to administrative data, we had to undertake primary data collection by way of an online survey, and substantial amounts of time were required to collate these data given the number of CRE affiliates and the level of activity. While the process was designed to be minimally intrusive, this data collection did create an additional administrative burden for researchers. As with any standard evaluation, impact assessments require resources to collect, analyse and report the evidence. As such, impact assessment may not be feasible for a CRE that has not allocated a portion of its funding towards an impact assessment. FAIT has a function beyond identifying, analysing and reporting evidence of impact. It is a framework to actively encourage research translation to optimize the possible impact from funded research. This encouragement requires a focused resource. Consequentially, a substantial amount of the impact assessor role was spent educating and building capacity of CRE-Stroke research affiliates in impact-planning and research translation to get them on board with the exercise. In turn, this potentially contributed to optimizing the CRE’s impact.

Although limited in terms of the ability to monetize downstream impacts including changes in practice and potential improvements in care and health outcomes, FAIT was able to present a CCA of the ROI in CRE-Stroke, something rarely covered by other impact frameworks. The use of FAIT facilitated a wider range of reportable outputs and impacts from the AU$ 2.5 million direct investment by the NHMRC and AU$ 1.4 million in in-kind contributions. It stands to make a solid contribution to the understanding of potential benefits and returns from a major nationally competitive funding scheme in Australia, of which there has been only one other impact assessment [27]. With interest in research impact steadily growing, future assessments may allow the funder (NHMRC) to benchmark performance of various CREs and would provide the opportunity to assess the ROI in this funding scheme to assist with internal priority-setting.

The main areas of impact from CRE-Stroke were around capacity-building of the people involved, with different CRE-Stroke-supported initiatives to develop skills and expertise in clinician researchers, early career researchers, consumer representatives, policy-makers and consumers, consistent with the aims of a CRE [28]. The impact assessment was able to cover the areas in which capacities were built and the hours of capacity-building activity. More nebulous benefits such as the expansion of collaboration and growth in the CRE-Stroke network over time were difficult to measure without further investment in additional methodologies such as social network analysis.

### Limitations

Impact assessments are resource-intensive, and although the prospective collection of evidence is more cost-effective, not all the required data can be collected prospectively. Final metrics for the Payback assessment and data for the narratives and economic assessments were based on what could be feasibly collected. The lag between research translation and impact meant that valuations needed to be undertaken with reference to interim rather than final impacts.

We conducted this study in a real-world setting, which meant there were no controls (counterfactuals of what would have happened in the absence of CRE-Stroke); thus, attribution of impact was necessarily conservative and constrained, in some cases, by the evidence available to substantiate claims that specific impacts were attributable to CRE-Stroke. The boundaries between CRE-Stroke and other stroke rehabilitation activities were also blurred in many instances, making attribution challenging. Where appropriate and pragmatic, we relied on the CRE-Stroke members and affiliates themselves to determine the CRE-Stroke contribution and attribution to things like leveraged funding.

Although we scheduled regular monitoring and feedback which was coordinated across CRE-Stroke, the uptake was poor. This was mainly given the competing demands on researcher time. This limitation needs to be acknowledged for all subsequent applications of FAIT.

The direct monetized consequences from CRE-Stroke were limited because CRE-Stroke was mainly a research collaborative focused on capacity-building and translation, with few identified “interventions” being funded specifically from CRE-Stroke resources. The impact of the standardization of the research landscape created by the SRRR and the benefits of access to a stroke volunteer register and biobank are still being realized and are difficult to monetize. Hence, impact assessment as an evaluation technique for research investment must acknowledge that the assessment is capturing a point-in-time snapshot of a research story that continues to unfold.

## Conclusion

This case example in the developing field of impact assessment illustrates how researchers can use evidence to demonstrate and report ROI in a way that is understood by funders and the broader community. Our impact assessment of CRE-Stroke involved the prospective measurement of nontraditional but core features of a CRE such as capacity-building and research translation, along with traditional impact measures such as research outputs and citations. We were able to demonstrate that CRE-Stroke acted as intended, in that it brought collaborators together to create something bigger than the sum of its parts. Further, through application of FAIT, we have quantified that CRE-Stroke generated a substantial return on the investment. Our methods can be used by other research teams to provide comprehensive evidence to governments and other research funders about what has been generated from their research investment.

### Supplementary Information

**Additional file 1.** Research impact survey questionnaire.

## Data Availability

The datasets used and/or analysed during this study will be available from the corresponding author on reasonable request after the publishing of the results.

## Appendix

### CRE-Stroke capacity-building activities

**Table:**

Date	Capacity-building activity
6 May 2015	Hunter Stroke Fatigue Forum, Toby Cummings, Jude Czerenkowski, Andrew Clarkson, Rohan Walker, Michael Hensley, Gillian Mead, Neil Spratt & Michael Pollack, HMRI Newcastle
17 October 2015	Stroke Rehabilitation Practice and Research Workshop, AFRM/NZRA conference, Wellington, NZ
1 February 2016	3rd Optimising Health Environments Forum
26 February 2016	CRE Seminar—Topic unknown
29 April 2016	CRE Seminar—Neuroimaging measures of recovery in stroke
9 May 2016	Stroke Recovery and Rehabilitation 1st Roundtable, Philadelphia, USA
27 May 2016	CRE Seminar—Novel interventions for upper limb recovery
24 June 2016	CRE Seminar—Exercise prescription after stroke
13 July 2016	Seminar on Rehabilitation and Stroke Recovery: Insights from Animal Models by Dale Corbett, Florey, Melbourne
14 July 2016	Pre-conference Rehabilitation Workshop at APSC, Dale Corbett, Julie Bernhardt, Maria Crotty, Claire Morris, Caitlin Brandenberg, Kevin Hill, Rachel Wenke, Peter New & Natasha Lannin, Brisbane
29 July 2016	CRE Seminar—Linked Data: What is it and how can it be used research?
26 August 2016	CRE Seminar—Move more, sit less. A new target for secondary stroke prevention?
9 September 2016	Industry Roundtable, Julie Bernhardt & Michael Nilsson, Florey, Melbourne
9 September 2016	CRE Stroke Rehabilitation Seminar, Liz Lynch, Nadine Andrew & Di Marsden, Florey, Melbourne
9 September 2016	CRE Seminar—5-min Research Snapshot comp, Implementation in stroke rehab, Exploring the value of data linkage
14 November 2016	Clinical Trials Development and Management Workshop, CRE Faculty, Florey, Melbourne
25 November 2016	CRE Seminar—ATTEND trial results
20 February 2017	4th Optimising Health Environments Forum
24 February 2017	HMRI Seminar—CRE Stroke Rehab and Brain Recovery Seminar- EXaCTT project
9 March 2017	Seminar—Improving design of dissemination and implementation strategies to promote evidence-based care
31 March 2017	CRE Seminar—Fitness training early after stroke
28 April 2017	CRE Seminar—Individual patient modelling based on AVERT data
26 May 2017	CRE Seminar—MiDAS (Modafinil in Debilitating Fatigue After Stroke): Primary trial results & an overview of secondary analyses
7 June 2017	CRE Seminar- Open Session: Implementing Evidence into Practice in The Age of “Alternative Facts”—Mark Bayley, and Supporting the Health and Medical Research Continuum—Peter Thomas, HMRI, Newcastle **(I combined with J (same conference)**
8 June 2017	CRE Newcastle Forum—CRE Mid-Term Overview and the National Vision, Julie Bernhardt, Michael Nilsson, Andrew Searles & Michael Pollack, HMRI Newcastle
8 June 2017	CRE Networking Dinner, Newcastle
9 June 2017	Early – Mid Career Implementation Research Workshop—Mark Bayley, Elizabeth Lynch & Shanthi Ramanathan, HMRI, Newcastle
30 June 2017	CRE Seminar—Environmental Enrichment
28 July 2017	CRE Seminar—Enhancing treatment fidelity for behavioural therapy in stroke rehabilitation in a complex clinical trial: The Very Early Rehabilitation in Speech (VERSE) experience
22 August 2017	Pre-Conference Rehabilitation Workshop at SSA Conference, Anna McRae & Erin Godecke, Queenstown, NZ
11 September 2017	Clinical Trials Development and Management Workshop 2017, CRE Faculty, Florey, Melbourne
13 September 2017	Masterclass with PhD students and Postdocs, Steven Cramer, Florey, Melbourne
13 September 2017	Seminar—Brain Repair after Stroke—Steven Cramer, Parkville, Melbourne
13 September 2017	Florey Seminar—Brain repair after stroke
15 September 2017	HMRI Seminar—Brain repair after stroke impact and health technology assessment in Health Care—what is relevant for rehabilitation medicine?
20 September 2017	CRE sponsored breakfast session at RMSANZ
13 October 2017	Media Training Workshop—Linda Drummond, Emma Beckett & Carmen Lahiff Jenkins, HMRI Newcastle (Webinar with Florey, Melbourne)
24 October 2017	Consumer Forum—What will make this research program different, compelling, and impactful? facilitated by Leigh Gassner and Adrian O’Malley, Docklands, Melbourne
27 October 2017	CRE Seminar—SRRR
23 February 2018	CRE Seminar—Clinical practice guideline development for early stroke rehabilitation
14 March 2018	CRE Seminar—Neural substrates of CNS recovery
19 March 2018	5th Optimising Health Environments Forum
27 April 2018	CRE Seminar—Advanced imaging of stroke in patients with sensory impairments
2 May 2018	Actively planning for research translation—Maximising benefits for researchers and end users—Elizabeth Lynch and Shanthi Ramanathan presented at National Stroke Data and Quality Workshop, Parkville, Melbourne
2 May 2018	HMRI Seminar—Spotlight on Stroke
25 May 2018	CRE Seminar—Upper limb motor recovery after stroke
31 May 2018	CRE Seminar—Stroke: using stem cell derived human neurons to find new drugs for human targets
27 June 2018	ECR Networking dinner in conjunction with Grant Development Workshop, Werribee, Melbourne
28 June 2018	Grant Development Workshop (2 days), Werribee, Melbourne
26 July 2018	Actively planning for research translation—Maximising benefits for researchers and end users—Elizabeth Lynch and Shanthi Ramanathan, HMRI, Newcastle in conjunction with Hunter Cancer Research Alliance
7 August 2018	Pre-Conference Rehabilitation Workshop at Stroke 2018 Bridging the Continuum, Convention Centre, Sydney
7 August 2018	ECR Networking Dinner, Sydney
31 August 2018	CRE Seminar—Detecting and preventing cognitive decline associated with ageing and vascular health
21 October 2018	Stroke Recovery and Rehabilitation 2nd Roundtable, Montreal, Canada
30 November 2018	Impactful CV workshop—Shanthi Ramanathan, Florey, Melbourne
30 November 2018	Seminar Using robotic technology to better understand and enhance stroke recovery—Florey, Melbourne and (Webinar@HMRI); and Young Stroke Research Forum, Florey Melbourne
22 February 2019	CRE Seminar—Making the most of your scientific publications
29 March 2019	CRE Seminar—Stroke Telemedicine Update
7 May 2019	Clinical Trials Development and Management Workshop

CRE: Centre of Research Excellence; HMRI: Hunter Medical Research Institute; AFRM/NZRA: Australian Faculty of Rehabilitation Medicine / New Zealand Rehabilitation Society; APSC: Australian Public Service Commission; ATTEND: Family-led Rehabilitation after Stroke in India; EXACT: Effect of Lower vs Higher Oxygen Saturation Targets on Survival to Hospital Discharge; AVERT: A Very Early Rehabilitation Trial; MIDAS: Modafinil in Debilitating Fatigue after Stroke; VERSE: Very Early Rehabilitation in Speech; SSA: Smart Strokes Australia; NZ: New Zealand; RMSANZ: Rehabilitation Medicine Society of Australia and New Zealand; SRRR: Stroke Rehabilitation Research Roundtable; CNS: Central nervous system; ECR: Early career researcher; CV: curricullum vitae

## Abbreviations

AU$: Australian dollar
CRE: Centre for Research Excellence
CRE-Stroke: Centre for Research Excellence in Stroke Rehabilitation and Brain Recovery
FAIT: Framework to Assess the Impact from Translational health research
NHMRC: National Health and Medical Research Council
ROI: Return on investment
S.A: Sensitivity analysis
