# Supplementary material for: Impact assessment of the Centre for Research Excellence in Stroke Rehabilitation and Brain Recovery
Source: Health Res Policy Syst. 2023 May 1;21:30. doi: 10.1186/s12961-023-00974-y (PMC10152619; doi:10.1186/s12961-023-00974-y)
Supplement: Supplementary file 1 — Additional file 1. Research impact survey questionnaire. [file 12961_2023_974_MOESM1_ESM.pdf]

NHMRC CENTRE OF RESEARCH EXCELLENCE
Stroke Rehabilitation and Brain Recovery

Additional file 1: Research impact survey

Section 1: Demographics and clinical/research roles

Q1 Which category best describes you? :

- ☐ Early-career researcher (8 years post PhD)
- ☐ Mid-career researcher (8 to 15 years post PhD)
- ☐ Senior researcher (more than 15 years post PhD)
- ☐ PhD/doctoral student
- ☐ Clinician researcher
- ☐ Other (e.g., policy maker, industry representative, consumer, undergraduate student):

____

Q2 What is your age?

- ○ 18-30
- ○ 31-40
- ○ 41-50
- ○ 51-60
- ○ 61+

Q3 What is your gender?

- ○ Female
- ○ Male
- ○ Other
- ○ Prefer not to say

Q4 How would you describe your main geographical work/study location?

- ◯ Metropolitan
- ◯ Regional
- ◯ Rural/remote

Q5 What is your professional background/qualification/area of study? (tick all that apply):

- ☐ Clinical/Health Psychology
- ☐ Clinical Neuropsychology
- ☐ Medicine. Please add specialty: ____
- ☐ Nursing
- ☐ Nutrition/Dietetics
- ☐ Occupational Therapy
- ☐ Paramedicine/Ambulance service
- ☐ Pharmacy
- ☐ Physiotherapy
- ☐ Social work
- ☐ Speech Pathology
- ☐ Other: ____

Q6 How long have you been involved with CRE-Stroke?:(The CRE was established in Nov 2014)

- ☐ Less than 6 months
- ☐ 6 months to 1 year
- ☐ 1-2 years
- ☐ 3-4 years
- ☐ Over 4 years.

Q7 Which of the above CRE-Stroke organized activities have you attended and how many of each (tick relevant cells/ ignore shaded cells)?

	0	1	2	3-5	6-9	10+
Implementation workshop						
Grant writing workshop						
Clinical trials workshop						
Research translation workshop						
Rehab practice and research						
ECR Media workshop						
Twitter Workshop						
Impactful CVs workshop						
Rehab Day @ SSA/APSO/Florey						
Forums [Consumer/Fatigue/Young Stroke Survivor]						
June 2017 Newcastle Forum & mid-term review						
Seminars [Insights from animal models, Evidence into practice, Supporting HMR continuum, Brain repair after stroke, Robotic technology in rehab, Making the most of your publications]						
Friday Teleconferences						
Shut up and write sessions						
ECR Networking dinner						

Q8 Of all the activities you have attended, please pick up to five of them where you were able to implement something you learnt to your research.

Please list these here:

1. ____

2. ____

3. ____

4. ____

5. ____

NHMRC CENTRE OF RESEARCH EXCELLENCE
Stroke Rehabilitation and Brain Recovery

Section 2: Impact of activities

Activity 1

Q9 Would you say that participating in Activity 1 had ____ impact on your research or the way you conduct your research?

- ◯ No (skip next question)
- ◯ A little
- ◯ Some
- ◯ Moderate
- ◯ Significant
- ◯

Q10 How did you apply the knowledge/skills/ideas gained from Activity 1? Describe what you did using an example:

____

____

____

____

____

Q11 Did this application make any difference to you or your research? Did it change any outcome or contribute to any outcome?

1. Yes
2. No (skip Q12)

Q12 Describe what sort of impact you achieved from applying the skills/knowledge/ideas you learnt from Activity 1. Was it greater translation of your research? grant success? better clinical trial systems? set up and development of a Twitter profile?

____

____

____

____

____

____

____

(Repeat Q9-12 for each of the activities 1-5)

Thank you very much for completing this survey!
